# Impact of stereotactic radiosurgery dose on local control and radiation necrosis in metastatic brain lesions: a comparative study

**DOI:** 10.3389/fonc.2026.1894130

**Published:** 2026-08-03

**Authors:** Mohammad Mukahal, Atef F. Hulliel, Adam Abdallah, Abdullah Alzibdeh, Tariq Alrawajih, Hadeel Asfour, Ala’a Khanfar, Haneen M. Suleiman, Maysa Al Hussaini, Nisreen Amayiri, Mohammad Alsmairat, Abdelatif Almousa, Nasim Sarhan, Shatha Abu Taha, Ahmad K.H. Ibrahimi

**Affiliations:** 1 Department of Radiation Oncology, King Hussein Cancer Center, Amman, Jordan; 2 Faculty of Medicine, Jordan University of Science & Technology, Irbid, Jordan; 3 Department of Neurosurgery, King Hussein Cancer Center, Amman, Jordan; 4 Primary Care, Ministry of Health, Amman, Jordan; 5 Pulmonary and Critical Care, Chicago Ridge Medical Center, Chicago, IL, United States; 6 Department of Research, King Hussein Cancer Center, Amman, Jordan; 7 Department of Pathology, King Hussein Cancer Center, Amman, Jordan; 8 Department of Pediatrics, King Hussein Cancer Center, Amman, Jordan

**Keywords:** local control, metastatic brain lesions, radiation, radiation necrosis, stereotactic radiosurgery

## Abstract

**Purpose:**

This study aimed to evaluate and compare the efficacy of stereotactic radiosurgery (SRS) prescription doses on local control (LC) rates of brain metastases and radiation necrosis (RN) risk.

**Methods:**

A retrospective review was conducted on patients treated between January 2018 and December 2023 at a single institution with single-fraction SRS at doses of 20 Gy, 22 Gy, or 24 Gy. The effects of radiation dose on LC and RN were evaluated using univariate analysis, then with multivariate logistic regression adjusting for gross tumor volume (GTV) and history of post-SRS whole-brain radiation therapy (WBRT).

**Results:**

The study included 332 brain metastases from 159 patients. 127 lesions were treated with 20 Gy (38.3%), 183 with 22 Gy (55.1%), and 22 with 24 Gy (6.6%). Median follow-up was 14 months. One-year LC rates were 96.3% for 22 Gy versus 90.1% for 20 Gy (p = 0.032). On multivariable logistic regression, 22 Gy was still associated with higher odds of 1-year LC compared with 20 Gy (adjusted OR 3.04, 95% CI 1.22–7.59; p = 0.017). Both SRS dose and GTV were independent predictors of RN (dose adjusted OR 2.05, p = 0.016; GTV adjusted OR 1.40 per doubling volume, p < 0.001). Overall survival did not differ significantly by dose (p = 0.63).

**Conclusion:**

In this retrospective cohort, SRS at 22 Gy was associated with higher local control than 20 Gy. Larger GTV volumes were independently associated with a higher risk of RN. WBRT post SRS was not an independent risk factor for RN.

## 1 Introduction

Brain metastases (BMs) represent the most common type of intracranial malignancy, significantly surpassing the incidence of primary brain tumors. They are observed in approximately 20% to 40% of all cancer patients (1–3). Without therapeutic intervention, the prognosis for patients with BMs is poor, with median survival typically measured in weeks (4). However, with optimal treatment strategies, median survival can extend from 3 to 18 months (5).

Radiation therapy is central in the management of BMs. Historically, whole-brain radiation therapy (WBRT) was as the standard approach, particularly for diffuse metastases (1). Nevertheless, concerns regarding neurocognitive toxicity associated with WBRT have driven a shift towards the increased utilization of stereotactic radiosurgery (SRS) (3, 6).

The efficacy of SRS in achieving local control of brain metastases is well-documented, yet the optimal dosing strategies remain a subject of ongoing investigation. Studies have shown that local control rates are influenced by factors such as tumor size and histology, with larger metastases generally exhibiting poorer local control and an increased risk of necrosis following brain-directed radiation (7, 8). Internal dose escalation has been associated with increased local control, particularly for non-small cell lung cancer (NSCLC) brain metastases (8). However, the idea of better local control through higher radiation doses must be carefully balanced against the risk of radiation necrosis (RN), a significant cause of morbidity (9).

Radiation necrosis is a critical consideration in SRS planning, with several factors contributing to its incidence. Research indicates that larger tumor diameter and higher radiation doses are consistently associated with an increased risk of RN (9, 10). Therefore, a comprehensive understanding of the relation between SRS dosing, local control, and the risk of radiation necrosis is essential for optimizing patient outcomes. This comparative study aims to investigate the impact of varying SRS doses on local control and radiation necrosis in metastatic brain lesions, providing further insights into dose-volume relationship in SRS.

## 2 Materials ad methods

This study was conducted and reported in accordance with the Strengthening the Reporting of Observational Studies in Epidemiology (STROBE) guidelines for cohort studies.

### 2.1 Study population

This retrospective study included patients who received SRS for metastatic brain lesions at our institution between January 2018 and December 2023. Eligible patients were adults with radiologically confirmed, intact brain metastases from any primary malignancy, treated with single-fraction SRS at 20 Gy, 22 Gy, or 24 Gy, with available follow-up imaging for assessment of local control and radiation necrosis. Resection cavities and fractionated SRS plans were excluded. Exclusion criteria also included prior radiation treatment to the index lesion site (including prior SRS or WBRT to SRS) and lack of sufficient clinical or radiographic follow-up data. Post-SRS WBRT administered for subsequent intracranial progression was not an exclusion criterion.

Thirteen patients subsequently underwent surgical resection at some point after SRS, later in their clinical course. These patients were retained in the analysis given that their SRS treatment and follow-up data were complete and available.

### 2.2 Treatment delivery

All patients were treated with single-fraction SRS using a linear accelerator–based Brainlab system. Treatment planning was performed using high-resolution, contrast-enhanced MRI fused with CT simulation. Target volumes were delineated on MRI, and the planning target volume (PTV) was generated with institution-specific margins as appropriate.

Dose was prescribed to the PTV using standard conformity and dose gradient criteria, typically to an isodose line of 70–80%, in accordance with institutional practice. Coverage goals aimed for ≥95% of the PTV receiving the full prescription dose while respecting normal tissue constraints, with most lesions achieving more than 98% coverage.

SRS dose selection (20 Gy, 22 Gy, or 24 Gy) was determined at the discretion of the treating physician, guided by institutional protocols primarily incorporating lesion size, location, and proximity to critical structures. In general, higher doses (22–24 Gy) were preferred for smaller lesions remote from critical structures, while 20 Gy was used for larger lesions or those in proximity to eloquent structures such as the brainstem or optic apparatus, where dose de-escalation was warranted for safety. Single-fraction SRS was generally delivered for lesions <3 cm in maximum diameter; fractionated regimens were considered for larger lesions. WBRT was reserved for patients with more extensive intracranial disease burden or at physician discretion.

### 2.3 Follow-up and outcome assessment

Patients were followed with clinical evaluation and brain MRI at approximately 3-month intervals for the first 2 years after SRS, and every 6 months thereafter, or as clinically indicated.

Local control (LC) was defined as the absence of radiographic progression of the treated lesion on follow-up MRI, based on serial imaging assessment. Progression was determined by an increase in lesion size and/or new or increasing contrast enhancement consistent with tumor growth. For logistic regression, LC was used as a binary 1-year outcome (local control achieved vs. local failure within 12 months of SRS). For time-to-event analyses, LC was analyzed using Kaplan–Meier methods at the lesion level, with time measured from SRS date to the date of first documented local failure.

Radiation necrosis (RN) was diagnosed based on radiographic findings, including characteristic imaging features on MRI, advanced imaging modalities such as MR perfusion and spectroscopy. In selected cases, surgical pathology was used to confirm the diagnosis. Distinction between RN and tumor progression was made through multidisciplinary evaluation incorporating imaging, clinical course, and, when available, histopathology.

### 2.4 Statistical analysis

Differences in continuous lesion-level variables (notably GTV) across the three prescription-dose cohorts (20 Gy, 22 Gy, 24 Gy) were evaluated using one-way analysis of variance (ANOVA). Associations between dose group and binary outcomes (local control and radiation necrosis) were assessed in univariate analyses using χ² tests and univariate logistic regression to estimate crude odds ratios (ORs) with 95% confidence intervals (CIs).

Multivariate logistic regression models were constructed to evaluate the independent effect of SRS dose on (1) 1-year local control and (2) radiation necrosis. The primary model for local control included SRS dose (categorical), GTV (continuous, in cm³ and modelled on the log_2_ scale). The primary model for radiation necrosis included SRS dose, GTV, and history of post-SRS WBRT.

Kaplan–Meier methods with log-rank tests were used for time-to-event analyses of local control, distant brain failure, and overall survival.

To account for non-independence of multiple lesions within patients, cluster-robust (sandwich) standard errors and generalized estimating equations with an exchangeable correlation structure, clustered at the patient level with log2(GTV) as a covariate, were additionally estimated. All analyses were performed using SAS version 9.4 (SAS Institute Inc., Cary, NC).

## 3 Results

### 3.1 Patient demographics

A total of 159 patients with 332 brain metastases were included between January 2018 and December 2023. The cohort was balanced by sex (49.1% female, 50.9% male) with a median age of 55 years. Most patients (n = 146) underwent SRS as the sole local treatment; 13 patients subsequently underwent surgical resection later in the clinical course (timing and whether the resected lesion was the same SRS-treated lesion were not uniformly documented). Lesion progression was documented in 16 patients at the patient level; at the lesion level, 25 of 332 lesions (7.5%) experienced local failure (see Section 3.2).

Baseline performance status was favorable in nearly all cases (99.4% ECOG 0–2). The most common primary cancer was non-small cell lung cancer (NSCLC; 42.8%), followed by breast cancer (32.1%), small cell lung cancer (SCLC; 8.2%), melanoma (3.8%), and renal cell carcinoma (2.5%). Median follow-up was 14 months.

Extracranial disease was absent in 30.2% of patients, stable in 28.9%, progressive in 21.4%, and newly metastatic in 19.5%. Systemic therapy included chemotherapy (37.7%), targeted therapy (27.7%), combination regimen (24.5%) (see **Table 1**).

**Table 1:** A summary for patient findings (N patients = 159).

Variable	Frequency (%)
Gender	
Female	78 (49.1%)
Male	81 (50.9%)
Surgery (post-SRS, later in clinical course)	
No	146 (91.8%)
Yes	13 (8.2%)
At least one lesion progressed (patient level)	
No	143 (89.9%)
Yes	16 (10.1%)
Performance status (PS)	
ECOG 0, 1, 2	158 (99.4%)
ECOG 3 or 4	1 (0.6%)
Site of primary cancer	
NSCLC	68 (42.8%)
Breast	51 (32.1%)
SCLC	13 (8.2%)
Others	17 (10.7%)
Melanoma	6 (3.8%)
RCC	4 (2.5%)
Extracranial disease	
Absent	48 (30.2%)
Stable	46 (28.9%)
Progressive	34 (21.4%)
Recently became metastatic	31 (19.5%)
Type of systemic treatment^1^	
Chemotherapy	60 (37.7%)
Targeted therapy	44 (27.7%)
Targeted therapy, Chemotherapy	14 (8.8%)
Immunotherapy, Chemotherapy	9 (5.7%)
Targeted therapy, Chemotherapy, Hormonal	6 (3.8%)
Immunotherapy	5 (3.1%)
Targeted therapy, Immunotherapy	4 (2.5%)
Targeted therapy, Hormonal therapy	2 (1.3%)
Chemotherapy, Hormonal therapy	2 (1.3%)
Targeted therapy, Immunotherapy, Chemotherapy	2 (1.3%)
None	11 (6.9%)
Post-SRS WBRT^2^	
No	114 (71.7%)
Yes	45 (28.3%)
Patient status at last follow-up	
Alive	75 (47.2%)
Dead	84 (52.8%)
Number of lesions treated per patient	
1	65 (40.9%)
2	37 (23.3%)
3	17 (10.7%)
More than 3	40 (25.1.%)

^1^ Percentages for Type of Systemic Treatment are calculated at the patient level (N = 159 patients).

^2^ WBRT was administered after SRS for intracranial progression; prior WBRT to the index lesion site was an exclusion criterion.

NSCLC, non-small cell lung cancer; SCLC, small cell lung cancer; RCC, renal cell carcinoma; WBRT, whole-brain radiation therapy; ECOG, Eastern Cooperative Oncology Group.

At last follow-up, 75 patients (47.2%) were alive and 84 (52.8%) had died. Most patients had a limited number of lesions treated: 40.9% had a single lesion, 23.3% had two, 10.7% had three, the rest having more than three (25.1%).

Of 332 metastatic brain lesions analyzed, 127 (38.3%) received 20 Gy, 183 (55.1%) received 22 Gy, and 22 (6.6%) received 24 Gy. Radiation necrosis was identified in 69 lesions (20.8%), while the remaining 263 (79.2%) showed no evidence of necrosis. Distant brain failure occurred in 200 lesions (60.2%). Local failure was less frequent, affecting only 25 lesions (7.5%), whereas 307 (92.5%) maintained local control. The mean local control duration was 12.5 months (median 10.5, range 2–53, SD 8.7). GTV differed significantly across dose groups (ANOVA p < 0.001): mean GTV was 0.75 cm³ (median 0.30) for 20 Gy lesions, 0.33 cm³ (median 0.12) for 22 Gy lesions, and 0.41 cm³ (median 0.25) for 24 Gy lesions.

The slightly higher mean GTV in the 24 Gy group reflects its small size (n = 22) and a single larger lesion within the group.

### 3.2 Local control

**Figure 1:**
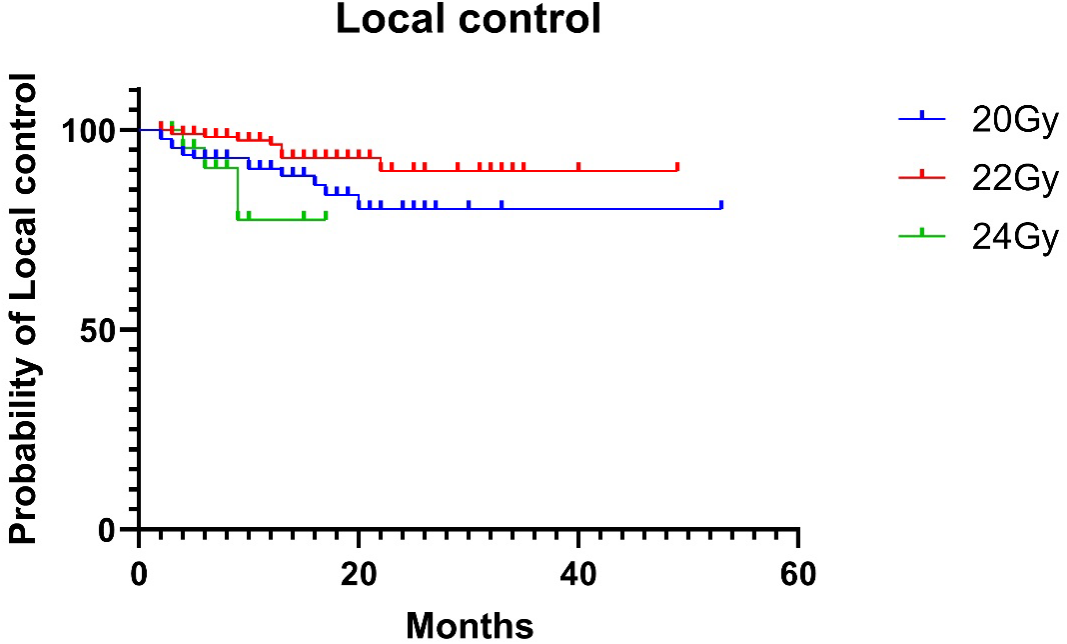
Kaplan–Meier curves for local control stratified by SRS dose (20 Gy, 22 Gy, 24 Gy). Analysis is at the lesion level (N = 332); comparisons are unadjusted. Log-rank p = 0.032. Because the 24 Gy group comprised only 22 lesions, comparisons involving this group should be interpreted as exploratory.

The overall one-year local control rate for the entire cohort was 93.3% (95% CI 89.9–96.1%). Kaplan–Meier estimated one-year LC rates by dose group were: 96.3% (95% CI 92.4–98.9%) for 22 Gy, 90.1% (95% CI 83.6–95.1%) for 20 Gy, and 86.5% (95% CI 65.2–98.6%) for 24 Gy (log-rank p = 0.032; **Figure 1**).

On univariate logistic regression (1-year binary LC outcome), 22 Gy was associated with significantly higher odds of local control compared with 20 Gy (OR 2.395, 95% CI 1.003–5.719; p = 0.049). After multivariate adjustment for GTV, 22 Gy remained significantly associated with higher local control (adjusted OR 3.044, 95% CI 1.221-7.592; p=0.0169There was no statistically significant difference in local control between 22 Gy and 24 Gy.

The association between 22 Gy and improved local control was directionally consistent and of similar magnitude under patient-level clustering (adjusted OR ≈ 3), remaining significant under cluster-robust standard errors (p = 0.044) but attenuated to non-significance under an exchangeable generalized estimating equations model (p = 0.23).Post-SRS WBRT was independently associated with reduced 1-year local control in the multivariate model (adjusted OR for LC: No WBRT vs. Yes WBRT 2.756, 95% CI 1.165–6.517; p = 0.021). On Kaplan–Meier analysis, one-year LC rates were 95.0% (95% CI 91.0–97.9%) without post-SRS WBRT versus 89.9% (95% CI 83.0–95.1%) with post-SRS WBRT (log-rank p = 0.009). WBRT was entered as an exploratory check in local control model.

### 3.3 Radiation necrosis

On univariable analysis, GTV was a significant predictor of radiation necrosis: each 1-cm³ increase in GTV was associated with increased odds of RN (OR 1.772, 95% CI 1.277–2.460; p = 0.001). Dose was also associated with RN on univariable testing: lesions treated with 20 Gy had higher odds of RN compared with those treated with 22 Gy (OR 2.495, 95% CI 1.428–4.360; p = 0.001). By dose group, RN occurred in 38 of 127 (29.9%) lesions treated with 20 Gy, 27 of 183 (14.8%) with 22 Gy, and 4 of 22 (18.2%) with 24 Gy (p = 0.004). The higher crude RN rate in the 20 Gy group reflects the significantly larger GTV in that cohort, after multivariable adjustment for GTV, higher SRS dose was associated with increased RN risk (see discussion).

In the multivariate model including SRS dose, GTV, and history of post-SRS WBRT, both dose and GTV remained independently associated with RN: dose (20 Gy vs. 22 Gy adjusted OR 2.053, 95% CI 1.145–3.680; p = 0.016) and GTV (adjusted OR 1.40 per doubling of volume, (95% CI 1.20–1.63; p < 0.001)”; The association between log2 (GTV) and radiation necrosis was robust to clustering.

Post-SRS WBRT was not significantly associated with RN in this multivariable model (adjusted OR 0.831, 95% CI 0.461–1.499; p = 0.539). The effect of systemic therapy class, including immunotherapy, on RN risk was not formally modeled in this study owing to small subgroup numbers and limited statistical power; this is acknowledged as a limitation.

ROC analysis identified GTV thresholds associated with RN risk. For 20 Gy lesions, a GTV cutoff of 0.325 cm³ (AUC 0.662) identified a higher-risk group: RN occurred in 42.6% (26/61) of lesions with GTV ≥ 0.325 cm³ versus 18.2% (12/66) of those with GTV < 0.325 cm³ (p = 0.002). For 22 Gy lesions, a cutoff of 0.203 cm³ (AUC 0.712) similarly discriminated risk: RN occurred in 26.9% (18/67) of lesions with GTV ≥ 0.203 cm³ versus 7.8% (9/116) of those with GTV < 0.203 cm³ (p < 0.001).

On bootstrap internal validation, discrimination was essentially unbiased (optimism-corrected AUC ≈ apparent), but the optimal cutpoints was unstable, ranging 0.10–0.38 cm³ (20 Gy) and 0.11–0.61 cm³ (22 Gy) rendering these cutpoints as investigational.

### 3.4 Survival analysis

**Figure 2:**
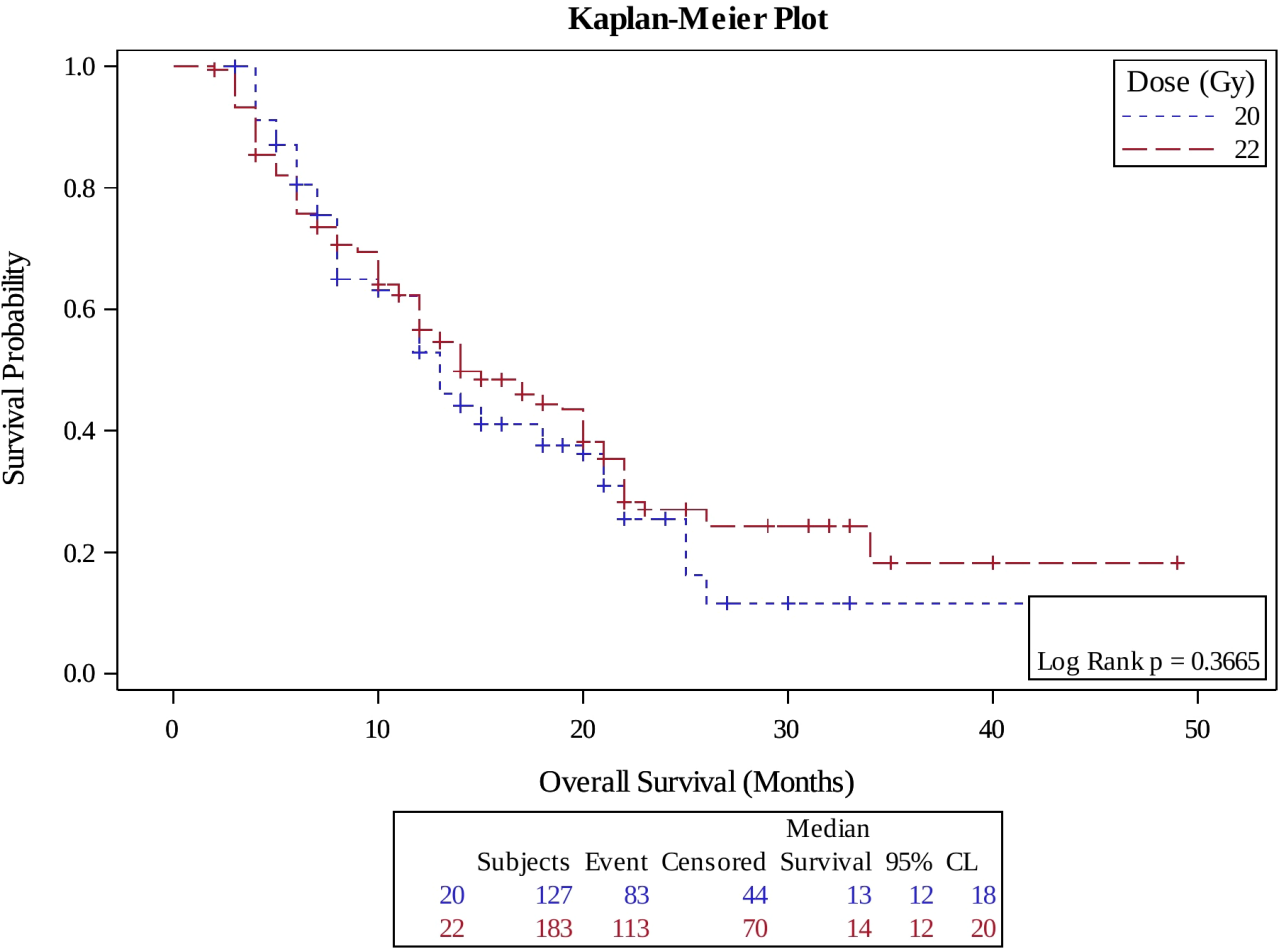
Kaplan–Meier overall survival curves stratified by radiation dose (20 Gy, 22 Gy, 24 Gy). Analysis is at the patient level. Log-rank p = 0.631. OS data are reported descriptively (log-rank result is reported for context, not as a formal causal claim).

**Figure 3:**
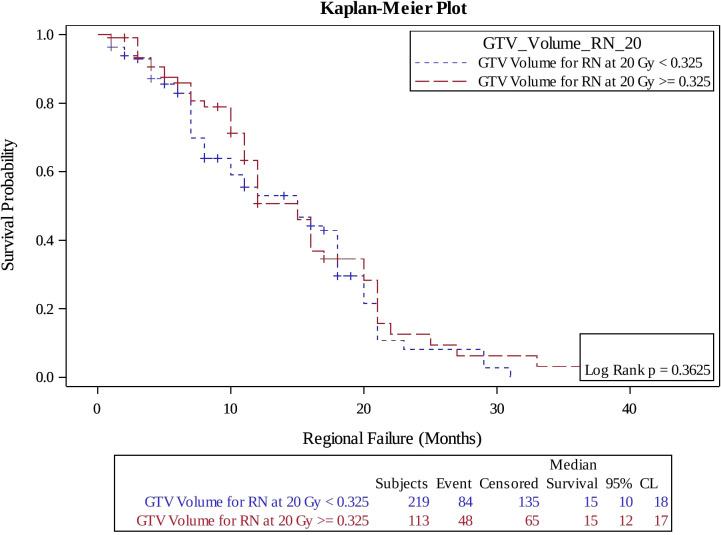
Kaplan–Meier overall survival (OS) curves stratified by GTV volume threshold for the 20 Gy subgroup.

**Figure 4:**
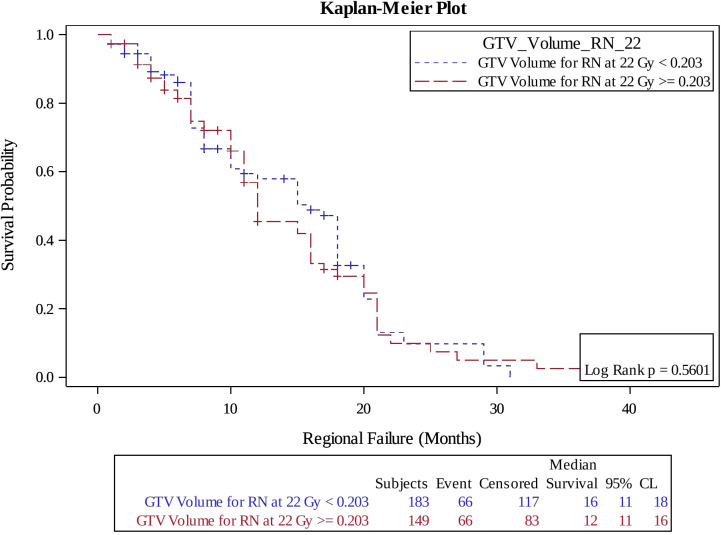
Kaplan–Meier overall survival (OS) curves stratified by GTV volume threshold for the 22 Gy subgroup.

For the entire cohort (N = 332 lesions; 159 patients), the median overall survival (OS) was 14 months, with a one-year OS rate of 55.1%. Kaplan–Meier curves stratified by radiation dose showed no significant differences in OS (log-rank p = 0.6307). The one-year OS rates were 52.9% (95% CI 43.8–62.0%) for 20 Gy, 56.6% (95% CI 49.1–63.9%) for 22 Gy (**Figure 2**), and 54.1% (95% CI 20.7–85.5%) for 24 Gy. OS data are reported descriptively; no causal relationship between SRS dose and survival was implied. GTV volumes at 20 Gy (**Figure 3**) and 22 Gy (**Figure 4**) for RN and LC subgroups generally did not affect OS, except for GTV volume at 20 Gy for LC, which showed a marginal difference (p = 0.044), though one-year OS rates were similar (54.8% vs. 55.4%).

Post-SRS WBRT, administered for subsequent intracranial progression, was reported in 45 patients (28.3%).

The median time to distant brain failure was 15 months, with a one-year rate of 52.0%.

## 4 Discussion

SRS is an essential treatment modality for BMs, offering a highly conformal and ablative radiation treatment that delivers high doses to tumor targets while sparing surrounding healthy brain tissue (1, 11). However, optimizing SRS dosing strategies remains an area of investigation, as the balance between achieving adequate local control and minimizing the risk of radiation necrosis is complex (7, 9). Factors such as tumor size, histology, and prior treatments significantly influence treatment outcomes and the incidence of complications (9, 12, 13).

The excellent overall 1-year local control rate of 93.3% observed in our cohort highlights the efficacy of SRS in managing brain metastases, consistent with contemporary literature (14, 15). Specifically, our multivariate analysis demonstrated that 22 Gy dose was associated with higher odds of local control compared to those receiving 20 Gy (adjusted OR 3.04; p = 0.017). This finding aligns with previous research suggesting a dose-response relationship for local control in SRS for brain metastases (7, 16). For instance, Vogelbaum et al. reported that a dose of 24 Gy to the tumor margin resulted in a significantly lower risk of local failure compared to 15 or 18 Gy, with a 1-year local control rate of 85% for 24 Gy versus 49% for 18 Gy (7). Other studies have explored optimal dosing, with recommendations ranging from 20–24 Gy for lesions under 2 cm (17). The concept of internal dose escalation has also been linked to improved local control, particularly for NSCLC brain metastases (8).

Retrospective series consistently showed similar outcomes. One analysis reported 1-year LC of 39.6% for lesions receiving ≤15 Gy, 71.7% for 16–20 Gy, and 92.3% for ≥21 Gy (18). Pooled analyses confirm this trend: for tumors ≤20 mm, 18 Gy gives ~85% 1-year control while 24 Gy yields ~95% (19). The landmark RTOG 95–08 randomized trial used 24 Gy (<2 cm) and 18 Gy (2–3 cm) SRS prescriptions and showed improved 1-year local control with SRS (82% vs. 71%) (20). Mohammadi et al. likewise confirmed that 24 Gy resulted in significantly better local control for metastases <2 cm and identified tumor size as an independent prognostic factor for local progression (16). Our finding that 22 Gy outperforms 20 Gy adds to this dose-response evidence. Other studies have emphasized the role of tumor histology, prior treatments, and systemic therapies in influencing local control rates (21, 22)as well as size as a predictor of reduced local control rates in larger lesions (13, 23).

No significant difference in local control was observed between 22 Gy and 24 Gy. However, the 24 Gy cohort comprised only 22 lesions, and therefore statically this comparison must be considered exploratory and underpowered. Null results cannot be interpreted as evidence of equivalence between these two dose levels.

Because post-SRS WBRT was delivered as salvage for intracranial progression, it is a consequence of disease progression rather than a baseline factor. We therefore excluded it from the primary local-control model and assessed it only in an exploratory analysis and its association with decreased local control reflects confounding by disease burden rather than a treatment effect, a concept that was also observed in other similar cohorts (24).

Radiation necrosis remains an important side effect; in a large Cleveland Clinic series of 896 patients encompassing 3, 034 brain metastases ≤2 cm, multivariable analysis demonstrated that lesions with diameter >1 cm (versus <1 cm) carried a higher risk of necrosis (adjusted RR ≈2.1) and a markedly greater risk of symptomatic necrosis (adjusted RR ≈4.8); tumor volume >0.1 cm³ was also a significant risk factor (16). Similarly, the University of Pittsburgh documented a 5-year cumulative incidence of neurologic complications of approximately 3% for targets ≤2 cm³, compared with 16% for those >2 cm³ (p = 0.0085) (25).

Radiation necrosis was identified in 20.8% of lesions in our cohort, consistent with published rates of 5–30% (1, 26, 27). The crude RN rates across dose groups appear paradoxical, as lower doses seemingly carried higher necrosis risk. This pattern is explained by confounding from tumor volume: lesions treated with 20 Gy had a substantially larger mean GTV (0.75 cm³ vs. 0.33 cm³ for 22 Gy), reflecting our institutional practice of prescribing lower doses to larger tumors. Therefore, multivariable adjustment for GTV was an essential step to carry, which showed dose-response relationship: higher SRS dose was independently associated with greater RN risk (20 Gy vs. 22 Gy adjusted OR 2.053, 95% CI 1.145–3.680; p = 0.016), while GTV independently contributed an approximately 40% increase in RN odds per doubling of volume (adjusted OR 1.40p < 0.001). These findings are consistent with established dose-volume predictors of radiation necrosis (28).

Notably, post-SRS WBRT was not independently associated with RN (adjusted OR 0.831; p = 0.539), and the direction of the estimate (OR<1) also supports the lack of causation. RN is primarily determined by the local SRS dose and target volume delivered at the time of treatment (29). Subsequent WBRT at standard palliative doses (20–30 Gy) is unlikely to increase the risk of radiation necrosis.

ROC analysis identified GTV thresholds that discriminate RN risk within each dose group: 0.325 cm³ for 20 Gy-treated lesions (AUC 0.662) and 0.203 cm³ for 22 Gy (AUC 0.712). For lesions below the ROC-derived GTV threshold of 0.203 cm³, RN occurred in only 7.8% of 22 Gy-treated lesions whereas for lesions exceeding this threshold, the RN rate rose to 26.9%. A parallel pattern was observed at 20 Gy, where lesions above the 0.325 cm³ threshold carried nearly 2.5 times the RN rate of smaller lesions (42.6% vs. 18.2%), these cutpoints were derived and tested in the same cohort, were unstable on bootstrap resampling (95% intervals ≈0.10–0.38 and 0.11–0.61 cm³), have only modest discrimination (AUC 0.66–0.71), and should be interpreted as hypothesis-generating pending prospective or external validation.

Despite this dose-dependent RN risk, dose escalation was not associated with decreased overall survival.

The median overall survival (OS) for the entire cohort was 14 months, with a 1-year OS rate of 55.1%, comparable to published series (7, 30). No significant differences in OS were observed across dose groups (p = 0.6307), consistent with the expected predominance of systemic disease as the driver of survival in brain metastasis patients (13).

This study is subject to several limitations. A retrospective design introduces inherent biases in data acquisition and lack of randomization. In this retrospective cohort, the prescribed dose was determined by the treating physician rather than by randomization and although GTV was adjusted for, other potentially relevant factors, such as anatomic location, were not. In addition, RN was diagnosed radiographically without blinding to dose, misclassification between RN and tumor progression cannot be entirely excluded. Dose-volume metrics, including V12Gy, a well-established predictor of RN, were not available as well as data on whether RN was symptomatic or asymptomatic and it’s subsequent RN-directed treatments. Lastly, the effect of systemic therapy class, particularly immune checkpoint inhibitors (ICIs), and tumor histology was not formally modeled owing to limited power and the mixed histological composition of the cohort. Notably, recent evidence suggests that concurrent or recent ICI use may amplify RN risk following SRS, potentially through enhanced T-cell-mediated perivascular inflammation (10, 31). As ICI use in brain metastasis patients continues to expand, future studies incorporating ICI timing, duration, and concurrent administration as covariates are needed to clarify this interaction.

## 5 Conclusion

In conclusion, our study found that 22 Gy is associated with superior local control compared to 20 Gy. GTV was identified as a significant independent predictor of radiation necrosis. Post-SRS WBRT was not independently associated with radiation necrosis. These findings contribute to the ongoing effort to optimize SRS protocols, balancing effective tumor control with minimizing treatment-related toxicities.

## Data Availability

The original contributions presented in the study are included in the article/supplementary material. Further inquiries can be directed to the corresponding author.
